# Overexpression of Bcl-2 promotes survival and differentiation of neuroepithelial stem cells after transplantation into rat aganglionic colon

**DOI:** 10.1186/scrt155

**Published:** 2013-01-16

**Authors:** Wei Liu, Weiming Yue, Rongde Wu

**Affiliations:** 1 Department of Pediatric Surgery, Provincial Hospital Affiliated to Shandong University, Jinan 250021, China; 2 Department of Thoracic Surgery, Qilu Hospital of Shandong University, Jinan 250014, China

## Abstract

**Introduction:**

Neural stem cell transplantation is a promising tool for the restoration of the enteric nervous system in a variety of motility disorders. However, limited cell viability after transplantation has restricted its regenerative capacity. The aim of this study was to evaluate the effect of transplantation of neuroepithelial stem cell (NESC) overexpressing anti-apoptotic gene Bcl-2 on the survival, differentiation and function of grafted cells in rat aganglionic colon.

**Methods:**

NESCs were isolated from neural tube of embryonic rat (embryonic day 11.5) and manipulated to overexpress the Bcl-2 gene. After transplantation into the benzalkonium chloride-induced rat aganglionic colon, grafted cells were visualized in colonic sections. Apoptosis and differentiation of the implanted cells were assessed 1, 4 and 8 weeks post transplantation, respectively. Eight weeks post transplantation, neuronal function of the colon was assessed by measuring the response of muscle strips to electrical field stimulation.

**Results:**

Transplantation with Bcl-2-NESCs reduced apoptosis within the transplant at 1 week compared with the vector-NESC grafted group. Our findings also indicated that overexpression of Bcl-2 in the transplanted NESCs enhanced differentiation into PGP9.5-positive and neuronal nitric oxide synthase-positive neurons at 8-week assessment. Moreover, electrical field stimulation-induced relaxation of colonic strips was also significantly increased in the Bcl-2-NESC grafted group.

**Conclusion:**

Transplantation of NESCs genetically modified to overexpress Bcl-2 may have value for enhancing survival and neurogenesis of grafted cells in the adult gut environment and for improving the efficacy of stem cell therapy following a broad range of gastrointestinal motility disorders.

## Introduction

Gastrointestinal motility disorders such as Hirschsprung's disease are characterized by complete or partial loss of neurons in variable lengths of the enteric nervous system (ENS) [1]. The treatment of the disorders is far from satisfactory and remains palliative at best. Theoretically, a real cure will restore or replace missing or dysfunctional neurons with healthy ones. Advances in molecular and stem cell biology have provided new avenues for therapy for ENS disorders and have led to the development of the ENS stem cell field [2-4]. Several potential sources of cells capable of generating enteric neurons have been explored for ENS replenishment in disorders characterized by dysfunctional or absent ENS including central nervous system-derived neural stem cells (NSCs), neural crest stem cells (NCSCs) and ENS progenitor cells [5-7]. During development, all neurons and glial cells of the ENS arise from NCSCs that migrate into and along the gut. Indeed, neuroepithelial stem cells (NESCs) isolated from the midembryonic rodent neural tube can differentiate into NCSCs. Using enteric neural precursors (that is, NESCs, NCSCs) therefore has the potential advantage of using cells of the same lineage as the desired phenotype. Our previous studies have also shown that transplantation of such NESCs resulted in the appearance of neuronal nitric oxide synthase (nNOS) and choline acetyltransferase-expressing neurons and improvements in colonic motility [8].

Although promising, post-transplant survival of NSCs represents a critical limiting factor for successful anatomical and functional repopulation of the host tissue. Transplanted NSCs can die from a variety of causes: physical injury, immune attack by the host, lack of trophic factors, or toxic environmental factors (free radicals, cytokines, and so on). A large portion of this cell death occurs as apoptosis within the first week after transplantation [9,10]. Augmenting neuronal replacement by enhancing the survival and maturation of endogenous progenitors is a potentially useful treatment for gastrointestinal neurodegenerative diseases. An alternative approach might involve the overexpression of an anti-apoptotic protein such as Bcl-2. The 26 kDa Bcl-2 anti-apoptotic protein belongs to the Bcl-2 family of proteins, which was originally found to be overexpressed in B-cell lymphoma [11]. The protein serves as a critical regulator of pathways involved in apoptosis, acting to inhibit cell death [12]. Increasing evidence suggests that, in addition to its anti-apoptotic properties, Bcl-2 has an important function in cell differentiation and growth. *In vivo *studies also indicated that Bcl-2 overexpression enhanced retinal axon regeneration after optic-tract transaction [13] and increased axonal growth of transplanted fetal dopaminergic neurons in the rat striatum [14]. We therefore aimed to determine whether overexpressing Bcl-2 of the transplanted NESCs in the gastrointestinal tract would improve cell survival and neuronal differentiation.

## Materials and methods

### Animals and surgery

All animal procedures were approved by the Guide for the Care and Use of Laboratory Animals published by the National Institute of Health (NIH publication No 85-23, revised 1985). Denervation procedure was performed on 12-week-old female Wistar rats. Topical application of benzalkonium chloride, a cationic surfactant agent, damages nerve elements selectively, leaving other tissues intact [15]. The enteric plexus of rat colon was eliminated by serosal application of 0.5% benzalkonium chloride (Sigma, St Louis, MO, USA) that has been successfully employed in our previous work [8].

### Isolation and culturing of rat neuroepithelial stem cells

Cell culture reagents were obtained from Invitrogen (Carlsbad, CA, USA). Briefly, trunk segments of embryonic day 11.5 Wistar rats were isolated in a dish containing cold Hank's buffered salt solution. Gentle trituration was employed to separate neural tubes from the somites. Tubes were dissociated using a 0.05% trypsin/ethylenediamine tetraacetic acid solution for 5 minutes at 37°C. After digestion, a cell suspension was obtained and resuspended in neurobasal medium containing B27, plus 20 ng/ml basic fibroblast growth factor. Cells were grown as free-floating clusters (neurospheres). The spheres were maintained at 37°C with 95% air and 5% CO_2 _and were passaged by mechanical dissociation every 5 to 7 days.

### Genetic modification of neuroepithelial stem cells

pcDNA3.1/GFP, pcDNA3.1/Bcl-2, or pcDNA3.1 (Invitrogen) was used for transfection. NESCs at passage 3 were trypsinized and washed. Approximately 1.5 × 10^7 ^cells were transfected with 10 μg linearized plasmid and 2 μg circular pKO Select neo (Stratagene, La Jolla, CA, USA). Briefly, NESCs were suspended in buffer (20 mM HEPES, 137 mM NaCl, 5 mM KCl, 0.7 mM Na_2_HPO_4_, 6 mM dextrose, pH 7.05) and electroporated in a BioRad Gene Pulser (0.4 cm gap electrode at 230 V and 960 μF). After electroporation, cells were plated and cultured. Determination of transfection efficiency was performed 24 hours after transfection by fluorescence microscopy (Olympus, Tokyo, Japan). For each experiment, at least three microscopic visual fields were counted, and the ratios of GFP-expressing cells to nonfluorescent cells were calculated. NESCs transfected with pcDNA3.1/Bcl-2 and pcDNA3.1 were termed Bcl-2-NESC and vector-NESC, respectively. The Bcl-2 protein expression level was evaluated by western blotting. All experiments and cell number determinations were performed in triplicate. Cell cultures for transplantation were checked for viability by trypan blue assay and viability was always > 90%.

### Cell transplantation

Four weeks after the denervation procedure, we performed cell transplantation. Animals were divided into Bcl-2 (Bcl-2-NESC transplantation) and control (vector-NESC transplantation) groups. Rats received daily immunosuppression with cyclosporine A (15 mg/kg, intraperitoneally; Novartis Pharmaceuticals, Cambridge, MA, USA) initiated 3 days prior to transplantation. Cells were pre-labeled with 4',6-diamidino-2-phenylindole (DAPI; Sigma) 1 hour before transplantation. After washing with PBS, labeled NESCs suspended in PBS were injected into the denervated colonic wall surgically from the serosa (100 μl; 5 × 10^6 ^viable cells per rat). Cells were slowly injected and the capillary was fully retracted 5 minutes after injection to avoid reflux of cells. The sites of injection were labeled with 6-0 suture. Animals were sacrificed at 1, 4 and 8 weeks post transplantation. Cell apoptosis was examined at 1 and 4 weeks and cell differentiation was evaluated at 8 weeks. At the end of the observation period, the treated colons were removed, washed with PBS and snap frozen in liquid nitrogen. Frozen sections embedded in optimum cutting temperature medium (12 μm in thickness) were prepared. An Olympus BX60 microscope (Olympus) was used to examine the sections and acquire the images. The neuronal function was assessed by measuring the responses of colonic strips in an organ bath in response to electrical field stimulation (EFS) at 8 weeks.

### Western blotting

Western blotting analysis was performed *in vitro *or 1, 4 and 8 weeks after cell transplantation to measure Bcl-2 protein expression. Cell and colon (longitudinal and circular muscles with adherent enteric plexus) extracts were washed three times with PBS and subsequently were homogenized in ice-cold lysis buffer, containing 2% SDS, 100 μmol proteinase cocktail inhibitor, 1 mmol phenylmethyl sulfonylfluoride, 1 mmol dithiothreitol, and 5 mmol ethylenediamine tetraacetic acid in 50 mmol Tris-buffered saline (50 mmol Tris-HCl; pH 7.4). After centrifugation (5 minutes, 12,500 × *g*), the supernates were diluted in four times concentrated Laemmli sample buffer. The protein content was determined (BSA Protein Assay Kit; Pierce, Rockford, IL, USA). For Bcl-2 analysis, samples (100 μg protein) boiled for 3 minutes were subjected to 10% SDS-PAGE. After electrophoresis, the proteins were transferred to nitrocellulose membranes (Bio-Rad, Hercules, CA, USA). The blots were incubated in blocking buffer (5% nonfat dry milk in Tris-buffered saline containing 0.1% Tween 20) for 1 hour at room temperature and probed overnight at 4°C with rabbit polyclonal anti-Bcl-2 antibody (1:1,000; Cell Signaling, Danvers, MA, USA) and rabbit polyclonal anti-β-actin (1:2,000; Santa Cruz Biotechnology, Santa Cruz, CA, USA) in blocking buffer. After washing in Tris-buffered saline-Tween 20, the blots were incubated with horseradish peroxidase-conjugated goat anti-rabbit IgG antibody (Sigma) at a dilution of 1:5,000 in blocking buffer for 1 hour at room temperature. The immunoreactive bands were visualized using enhanced chemiluminescence (ECL kit; Millipore, Billerica, MA, USA). The membranes were exposed to X-ray films. The intensities of the bands were quantified using the NIH Image 3.0 software. In all cases, β-actin was used as an internal standard.

### Apoptosis detection in neural grafts

Apoptotic cells in the transplant were identified by terminal uridine nick end-labeling (TUNEL) using the ApopTag kit (Oncor Inc., Gaithersburg, MD, USA). Cell death was quantified by counting the total cells labeled by DAPI and the percentage of TUNEL-positive cells. A stereological count of total cells and double-labeled cells were conducted on every 10th section to avoid repeated counting of the same cells.

### Immunohistochemical assessments

Colonic sections were double labeled with specific antibodies to identify the differentiated phenotype of the grafted cells. The sections rinsed in PBS were blocked in 10% goat serum for 30 minutes at room temperature and then incubated with primary antibodies solution at 4°C overnight. Enteric neurons were identified using polyclonal antibody against protein gene product 9.5 (PGP9.5, 1:1,000; ABCAM, Cambridge, UK), nNOS (1:1,000; Sigma) and choline acetyltransferase (1:1,000; ABCAM). Enteric glials were identified using a mAb against glial fibrillary acidic protein (1:1000; ABCAM). After washing, tissue was incubated for 30 minutes at room temperature with FITC (fluorescein)-conjugated goat anti-rabbit IgG (1:200; KPL, Gaithersburg, MD, USA) and TRITC (rhodamine)-conjugated goat anti-mouse IgG (1:200; KPL) and cover-slipped with a fluorescence mounting medium (Sigma). Primary antibody omission as well as primary antibodies preincubated with an excess of blocking peptides (ABCAM) served as negative controls, and no immunoreactivity was observed.

### Organ bath physiology

Animals were killed by cervical dislocation and the treated colons were removed and placed in Krebs buffer. The mucosa was removed and circular muscle strips (10 × 3 mm) were mounted between two L-shaped tissue hooks in 5 ml chambers containing Krebs buffer at 37°C and continuously bubbled with 95% O_2_/5% CO_2_. Tension was monitored with an isometric force transducer and recorded by a digital recording system (JH-2B; Instrument Company of Chengdu, Chengdu, China). Strips were stretched to 1 g (5 mN) and allowed to equilibrate for 30 minutes. The reactions were obtained by application of EFS (90 V, 5 to 40 Hz, 1 ms pulse for a duration of 5 minutes) in the absence or presence of tetrodotoxin (1 μmol/l; Sigma).

Comparisons among the groups (normal, denervation, control and Bcl-2) were performed by measuring the area under the curve of the EFS-induced reactions (AUC_R_) for 5 minutes and the baseline prior to the EFS for 5 minutes (AUC_B_) according to the following formula:

$$R={\text{AUC}}_{\text{R}}/){\text{AUC}}_{\text{B}}$$

### Statistical analysis

Mean values were reported together with the standard error of mean. Student's two-tailed *t *test was used for comparison of two experimental groups. Multiple comparisons were done using one-way analysis of variance followed by the Tukey test for multiple pairwise examinations. Changes were identified as significant if P < 0.05.

## Results

### Overexpression of Bcl-2 in genetically modified NESCs and grafts

**Figure 1:**
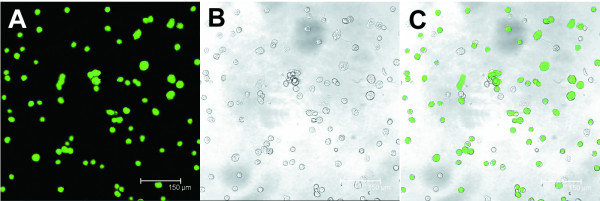
**Transfection of neuroepithelial stem cells with nonviral vector**. **(A) to (C) **Representative photomicrograph of neuroepithelial stem cells transfected with pcDNA3.1/GFP 24 hours after transfection. GFP-positive cells are shown in green. Scale bar = 150 μm.

**Figure 2:**
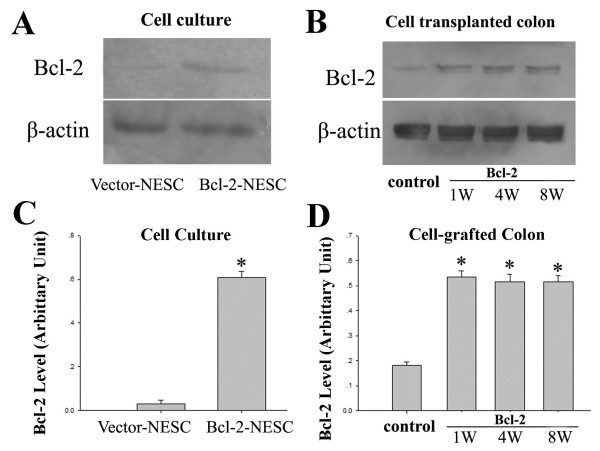
**Bcl-2 overexpression in cultured and transplanted cells**. **(A) **Significantly high expression of Bcl-2 was observed in cell cultures overexpressing the Bcl-2 gene 24 hours after transfection. In contrast, a weak expression of Bcl-2 was detected in vector-NESC. **(B) **In animal studies, 1 week after transplantation, a higher level Bcl-2 expression was seen in the transplanted colonic tissues from the Bcl-2 group compared with that from the control group. The Bcl-2 level remained high for up to 8 weeks after transplantation. **(C), (D) **Bar graphs showing the Bcl-2 levels in cultures and in cell grafted colons. At different days after transplantation, significantly higher Bcl-2 expression was seen in the colons of rats receiving Bcl-2-NESC than those receiving vector-NESC. *Significant difference compared with vector-NESC or control group (*P *< 0.05, *n *= 6 for cell culture experiments and *n *= 6 for animal studies). NESC, neuroepithelial stem cell.

The transfection efficiency for genetically modified NESCs was evaluated by pcDNA3.1/GFP as an internal control after 24-hour gene delivery. A representative GFP expression is shown in Figure 1. More than 85% of NESCs were transfected based on calculation analysis. NESCs overexpressing Bcl-2 contained higher levels of Bcl-2 protein than that in vector-NESC (Figure 2A, C). Western blotting showed elevated levels of Bcl-2 protein in the colons received Bcl-2-NESCs from 1 to 8 weeks after cell transplantation (Figure 2B, D).

### Bcl-2 overexpression decreased cell apoptosis in grafts

**Figure 3:**
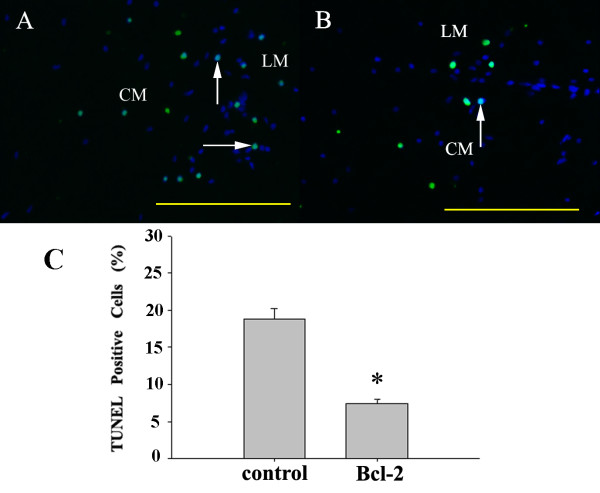
**Bcl-2 overexpression decreases cell death**. Representative pictures of neuroepithelial stem cell (NESC) grafts in cross-sections of rat colon in the **(A) **control group and **(B) **Bcl-2 group at 1 week post transplantation. 4',6-Diamidino-2-phenylindole-positive NESCs are shown in blue. Terminal uridine nick end-labeling (TUNEL) immunoreactivity is indicated in green. Transplanted NESCs that are TUNEL-positive are indicated by arrows. LM, longitudinal muscle; CM, circular muscle. Scale bar = 200 μm. **(C) **Quantitative analysis of the number of grafted NESCs that are TUNEL-positive in two groups. Data expressed as mean ± standard error of the mean; *n *= 6 for each group; *P < 0.05 versus control group.

To assess whether overexpression of Bcl-2 could play a role in preventing apoptosis in NESC, we performed TUNEL staining of colonic sections 1 and 4 weeks post grafting. Apoptotic cells were significantly reduced in the NESC grafts that were transfected with Bcl-2 when compared with the control group at 1-week assessment (18.8 ± 1.5% and 7.4 ± 0.6% TUNEL-positive cells for control and Bcl-2 groups, respectively, *P *< 0.05; Figure 3). Few TUNEL-positive cells were detected 4 weeks post transplantation and no differences were found between the groups (data not shown).

### Bcl-2 enhanced neuronal differentiation of NESC transplants

**Figure 4:**
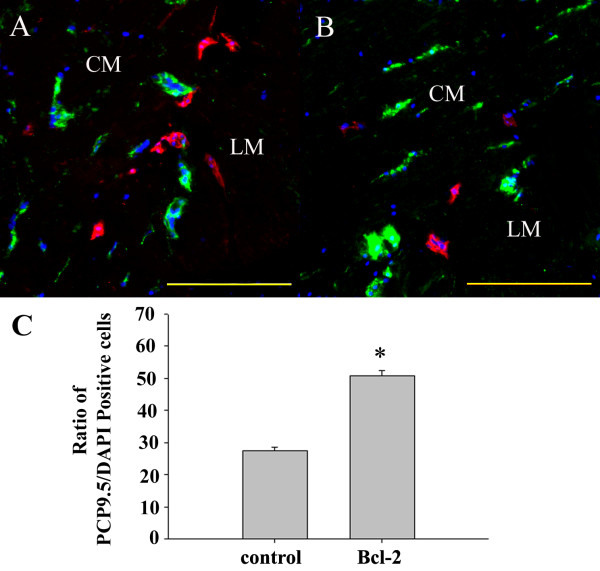
**Bcl-2 overexpression enhances the differentiation to PGP9.5-positive neurons of transplanted cells**. Representative pictures of the **(A) **control group and **(B) **Bcl-2 group in cross-sections of colon 8 weeks post transplantation. Grafted neuroepithelial stem cells are shown in blue. Protein gene product 9.5 (PGP9.5) immunoreactivity is indicated in green. Glial fibrillary acidic protein immunoreactivity is indicated in red. LM, longitudinal muscle; CM, circular muscle. Scale bar = 200 μm. **(C) **Ratio of PGP9.5-positive cells versus 4',6-diamidino-2-phenylindole (DAPI)-labeled cells was counted. Bcl-2 overexpression increased the ratio of PGP9.5-positive cells. *Significant difference compared with control group (*P *< 0.05, *n *= 6 for each group).

**Figure 5:**
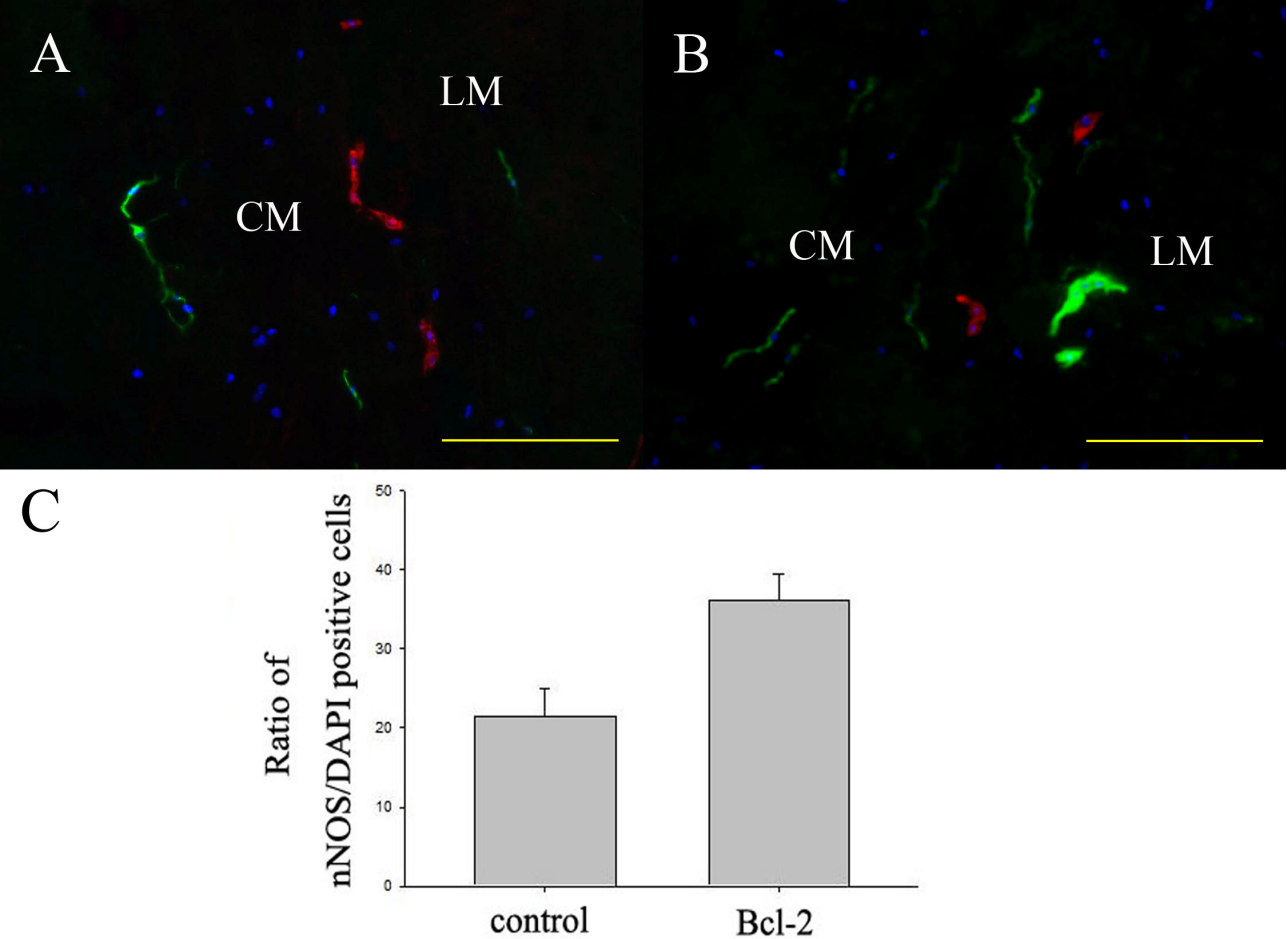
**Neuroepithelial stem cells offer enteric nervous system appropriate differentiation upon grafting into recipient aganglionic gut**. Evidence of 4',6-diamidino-2-phenylindole (DAPI)-labeled neuroepithelial stem cell graft (shown in blue) differentiation into neuronal nitric oxide synthase (nNOS; shown in green) and choline acetyltransferase (shown in red) neuronal subtypes was apparent at 8 weeks post transplantation in the **(A) **control group and **(B) **Bcl-2 group. LM, longitudinal muscle; CM, circular muscle. Scale bar = 200 μm. **(C) **Ratio of nNOS-positive cells versus DAPI-labeled cells was counted. Bcl-2 overexpression increased the ratio of nNOS-positive cells. *Significant difference compared with control group (*P *< 0.05, *n *= 6 for each group).

We next examined the possibility that Bcl-2 overexpression not only reduced cell death but also increased the neuronal differentiation. Eight weeks after transplantation, many transplanted cells were immunopositive for the neuronal marker PGP9.5. In addition to differentiating into neuron-like cells positive for PGP9.5, some transplanted cells in the colon stained positively for the enteric glia marker glial fibrillary acidic protein (Figure 4A, B). These markers co-localized with pre-labeled DAPI, confirming their transplant origin. Counted on the colonic sections of the control group at 8 weeks after transplantation, 29.1 ± 1.0% of transplanted vector-NESCs were PGP9.5-positive whereas 50.7 ± 1.8% of transplanted Bcl-2-NESCs were PGP9.5-positive (different at *P *< 0.05; Figure 4C). Furthermore, nNOS and choline acetyltransferase immunoreactivity was observed in host colon within transplanted NESCs (Figure 5A, B). Approximately 36.3 ± 1.3% of DAPI-positive NESCs expressed nNOS in the Bcl-2 group, a proportion apparently higher than that in the control group (21.4 ± 1.4%) (*P *< 0.05; Figure 5C).

### Bcl-2-engineered NESC transplantation improves relaxation of the colon

**Figure 6:**
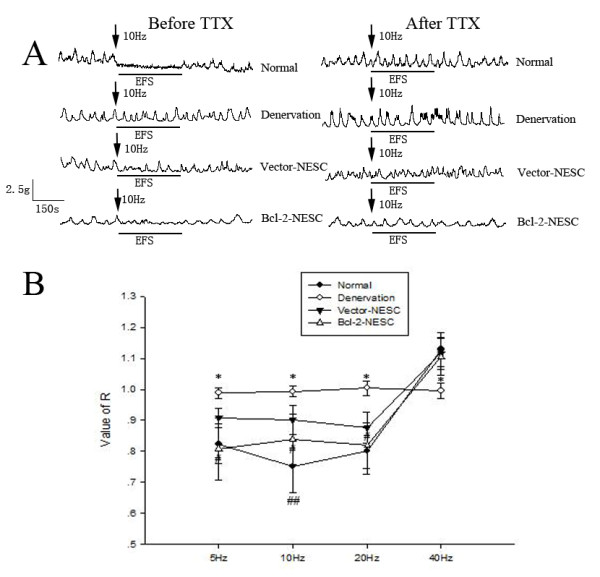
**Bcl-2 engineered neuroepithelial stem cell transplantation results in increased relaxation of the colonic muscle**. **(A) **Representative recordings showed the responses of the colonic circular muscle to electrical field stimulation (EFS) before and after incubation with 1 μmol/l tetrodotoxin (TTX) in four groups. ↓Marker of EFS (10 Hz) treatment. **(B) **Quantification of the value *R *at different frequencies in four groups. *Significantly different from normal, control and Bcl-2 groups. ^#^Significantly different from control group. ^##^Significantly different from Bcl-2 group with *P *< 0.05 (*n *= 8 for each group). NESC, neuroepithelial stem cell.

EFS of *ex vivo *preparations of the colon was performed to assess neurally mediated reactions of the muscle. As shown in Figure 6A, there was no reaction to EFS in denervated colon. In contrast, application of EFS resulted in a relaxation at lower than 20 Hz and a contraction at 40 Hz in colons of the normal, control and Bcl-2 groups. This effect was completely blocked by tetrodotoxin, confirming its neural origin. EFS-induced relaxation at three stimulating frequencies lower than 20 Hz was significantly increased in colonic muscle strips isolated from rats receiving Bcl-2-NESCs as compared with control groups according to the analysis of the value *R*. Significant differences between the control and Bcl-2 groups at stimulation frequencies of 5, 10 and 20 Hz are shown in Figure 6B (*P *= 0.004, *P *= 0.025 and *P *= 0.038, respectively). There was also a difference between the normal and Bcl-2 groups in EFS-induced relaxation merely at 10 Hz stimulation (*P *= 0.025). In addition, no significant difference of EFS-induced contraction in 40 Hz was found among normal, control and Bcl-2 groups.

## Discussion

Despite encouraging initial results of NSC replacement as a therapy for gastroenterology neurodegenerative diseases reported by many investigators, there are still significant limitations that prevent the clinical development of this therapy, including adequate survival, appropriate differentiation and physiological integration into the host tissue. Adequate survival of grafted cells is a problem of critical importance that will need to be addressed. Indeed, more than 90% of the grafted neurons usually die upon grafting, both in animal and human studies [16,17], and a large portion of this cell death occurs as apoptosis [9,10]. Adjunct measures to address this issue are therefore essential. Micci and colleagues reported only a very small proportion of transplanted central nervous system-derived NSCs actually could be found days to weeks after implantation for the restoration of the ENS. Using a selective inhibitor of caspase-1 (Ac-YVAD-cmk) at the time of central nervous system-derived NSC transplantation resulted in a significant improvement in graft survival 1 week post transplantation, but the number of grafted cells was drastically reduced at 2 and 4 weeks post transplantation [18]. This finding might suggest that the pharmacological treatment with a caspase inhibitor might not be sufficient in producing a long-lasting effect on graft survival. The present study therefore provided the first evidence that Bcl-2 overexpression by gene transfer reduced apoptosis of grafted cells in aganglionic colon of rat, enhanced differentiation into enteric neurons, and produced a further increase in functional benefits. In spite of the notion that Bcl-2 may represent a prototype for a new class of oncogenes [19] and overexpression of Bcl-2 is common in many types of human cancer [20], no tumors were seen in our study, perhaps reflecting the fact that the Bcl-2 overexpression did not completely prevent apoptosis in the host.

Many factors can contribute to post-transplant cell death, including necrosis, insufficient growth factor support, humoral and cellular immunity and programmed cell death, or apoptosis [21]. Immature cells are particularly vulnerable to apoptosis [22-25] and transplanted cells may die in large numbers. This death after transplantation can be an additional burden to the gut already compromised by a cellular debris load [26,27]. Our present studies revealed that genetic modification of NESCs with Bcl-2 effectively protected transplanted NESCs against apoptosis and increased cell survival 1 week after implantation. There were no drastic reductions in numbers of grafted cells at long-term assessment, possibly due to the fact that elevated Bcl-2 levels lasted from 1 to 8 weeks after cell transplantation.

The anti-apoptotic role of Bcl-2 has been well established in previous studies dealing with the differentiation of neural progenitors, and NSCs, in general [28,29]. Consistent with the idea that anti-apoptotic gene modifications may have beneficial effects on neural differentiation, Lee and colleagues reported that enhancing graft survival with the anti-apoptotic gene Bcl-X(L) could potentiate therapeutic benefits of NSC-based therapy for spinal cord injury [30]. After transplantation into the aged rat striatum, Bcl-X(L)-overexpressing hNS1 cells generated more neurons and less glia than the control ones, confirming the results obtained *in vitro*, which indicated an action of Bcl-X(L) modulating human NSC differentiation [31]. Our data also indicated that Bcl-2 overexpression in the transplanted NESCs resulted in promoting neuronal differentiation. We believed that the higher PGP9.5/DAPI ratio of NESCs with Bcl-2 overexpression was probably due to an increased neuronal differentiation from the transplanted cells.

We have also reported here that Bcl-2-engineered NESC transplantation in the aganglionic colonic region significantly improved EFS-induced relaxation compared with vector-NESCs. This effect most probably resulted from the restoration of neurally mediated relaxation by more differentiated neurons secreting important inhibitory enteric neurotransmitter in the Bcl-2 group, which was certified by immunohistochemistry analysis. Our results suggested that overexpression of Bcl-2 might selectively enhance the differentiation into nNOS-positive inhibitory enteric neurons of grafted cells. The finding that overexpression of Bcl-2 enhanced both the survival of transplanted cells and functional recovery supports the idea that the survival is therapeutically important to achieve, and further specifically indicates the value of apoptosis reduction through genetic manipulation.

In any case, the present study suggests that NESCs overexpressing Bcl-2 may be particularly useful for reducing apoptotic cell death, promoting neuronal differentiation and tissue functional recovery. Transplantation of gene-engineered NESCs may provide a novel and effective approach in the treatment of disorders of the ENS. However, further investigation will be needed to determine the possible mechanisms of differentiation and functional benefits of Bcl-2 expression in the transplanted cells. Moreover, the efficacy of the use of gene-modified cells and/or specific growth factors on the survival of NESC grafts also will be needed to be explored.

## Conclusion

In summary, we have confirmed that genetic modification with the anti-apoptotic Bcl-2 gene ameliorated cell survival and improved neuronal differentiation and function of grafted NESCs in aganglionic colon. Genetically engineering cells by Bcl-2 using a nonviral vector could be an effective strategy for increasing cell survival after cell transplantation. Transplantation of gene-engineered NESCs may provide a novel and effective approach in the treatment of disorders of the ENS.

## Abbreviations

AUC: area under the curve; DAPI: 4',6-diamidino-2-phenylindole; EFS: electrical field stimulation; ENS: enteric nervous system; GFP: green fluorescent protein; mAb: monoclonal antibody; NCSC: neural crest stem cell; NESC: neuroepithelial stem cell; nNOS: neuronal nitric oxide synthase; NSC: neural stem cell; PBS: phosphate-buffered saline; PGP9.5: protein gene product 9.5; TUNEL: terminal uridine nick end-labeling.

## Competing interests

The authors declare that they have no competing interests.

## Authors' contributions

WL participated in the design of the study, performed cell culture, genetic modification and the post-transplantation evaluation, and drafted the manuscript. WY carried out the establishment of animal model and cell transplantation, and performed the examination after implantation. RW conceived of the manuscript, participated in its design and coordination, analyzed and interpreted the data, and helped to draft the manuscript. All authors read and approved the final manuscript.
